# Prevalence and patterns of skin toning practices among female students in Ghana: a cross-sectional university-based survey

**DOI:** 10.1186/s13104-019-4327-8

**Published:** 2019-05-28

**Authors:** Williams Agyemang-Duah, Charlotte Monica Mensah, Reindolf Anokye, Esi Dadzie, Akwasi Adjei Gyimah, Francis Arthur - Holmes, Prince Peprah, Frimpong Yawson, Esther Afriyie Baah

**Affiliations:** 1 Department of Planning, Kwame Nkrumah University of Science and Technology, Kumasi, Ghana; 2 Department of Geography and Rural Development, Kwame Nkrumah University of Science and Technology, Kumasi, Ghana; 3 Centre for Disability and Rehabilitation Studies, Department of Health Promotion, Education and Disability, Kwame Nkrumah University of Science and Technology, Kumasi, Ghana; 4 Oxford Department of International Development, University of Oxford, Oxford, UK; 5 Sheffield Hallam University, Sheffield, UK

**Keywords:** Prevalence, Patterns, Skin toning practices, University students, Ghana

## Abstract

**Objective:**

The use of skin toning products has a deep historical background in low and middle-income countries. Yet, there is no empirical evidence on the prevalence, and patterns of skin toning practices among university students in Ghana. This study sought to examine the prevalence, patterns and socio-demographic factors associated with skin toning practices among female university students in Ghana using a sample of 389 undergraduate female students.

**Results:**

40.9% of respondents had practised skin toning within the last 12 months. Also, 51.3% used skin toning products such as creams (38.9%) and soap or gel (35.5%) to treat a skin disorder. Respondents aged 21 years were more likely to use skin toning products (AOR = 0.400, CI 0.121–1.320), those who had dark skin (AOR = 3.287, CI 1.503–7.187), attended public school (AOR = 1.9, CI 1.1–3.56) and those who attended girls school were more likely to use skin toning products (AOR = 10.764, CI 4.2–27.3). Furthermore, those who were in level 400 (AOR = 49.327, CI 8.48–286.9) and those receiving more than 500 cedis were also more likely to use skin toning products (AOR = 2.118, CI 0.419–10.703). Policy interventions that seek to reduce skin toning practices among university students should consider micro and broader socio-demographic factors.

**Electronic supplementary material:**

The online version of this article (10.1186/s13104-019-4327-8) contains supplementary material, which is available to authorized users.

## Introduction

Skin toning practice appears to have become a norm among people of various backgrounds, age, and gender [1–3]. Seeking for a lighter skin tone has always attracted attention in Western societies where fair or light skin colour has been a symbol of beauty, purity, sweetness, sex appeal, prominence as well as superiority and higher social ranking [4]. In Europe, White women have used bleaching creams to maintain radiant skin devoid of hyperpigmentation as a result of being exposed to heat [1] or the often-dreaded process of maturation [2].

Alghamdi [5] reported that the degree of skin toning practice has increased in Saudi Arabia, with an estimation of 38.9% reporting to be actively bleaching their skin [5]. Skin toning practices were reported among women in the Philippines [6], and in East Asia, skin toning practices have been reported among 30% Chinese, 20% Taiwanese, 18% Japanese and 8% Koreans [7].

In Africa, the World Health Organization claims that Nigeria has the highest percentage of women using skin toning products with reported 77% of women engaging in the practice [8]. A cross-sectional study in Togo reported that 58.9% of women used skin toning cosmetic products and that 30.9% used products containing mercury. Moreover, it has been reported that 25% of women in Bamako, Mali and 52% to 67% in Dakar, Senegal use skin toning products [9–12].

Skin toning practices have been reported among young women in Cameroon [12], and among 30% of women in Ghana [13]. Although the practice is global, African women are some of the biggest consumers of skin bleaching products, which include potentially harmful local concoctions made from household chemicals (e.g. automotive battery acid, bleach, laundry detergent, toothpaste), and over-the-counter creams, putting them at higher risk for a variety of adverse health outcomes [10]. In Ghana, data on skin toning practices among students remain primarily unavailable. The study, therefore, assesses the prevalence and patterns of skin toning practices and further examines socio-demographic factors associated with the practices among university students.

## Main text

### Methods

A cross-sectional University-based survey was conducted at the Kwame Nkrumah University of Science and Technology (KNUST) to examine patterns and prevalence of skin toning practices among female university students in Ghana. Being the second largest university in Ghana, KNUST is located in Kumasi and provides educational services for several people in Ghana and other neighbouring countries. This study recruited female undergraduate students from levels 100 to 400. Female students from the various colleges of the university such as Humanities and Social Sciences, Arts and Built Environment, Science, Health Science and Agriculture, and Natural Resources were selected using a two-stage cluster and random sampling techniques. Out of the 13,738 female students at KNUST, a formula by Miller and Brewer [14] were used to select 389 respondents as a representative sample size for the study.

$${\text{n}} = \frac{N}{{1 + N\left( {{\text{x}}^{2} } \right)}}$$

where n = sample size, N = total number of female undergraduate students in KNUST and x = margin of error.

$${\text{n}} = \frac{13{,}738}{{1 + 13{,}738\left( {0.05^{2} } \right)}}$$

n = 388.682 or approximately 389 respondents.

**Fig. 1:**
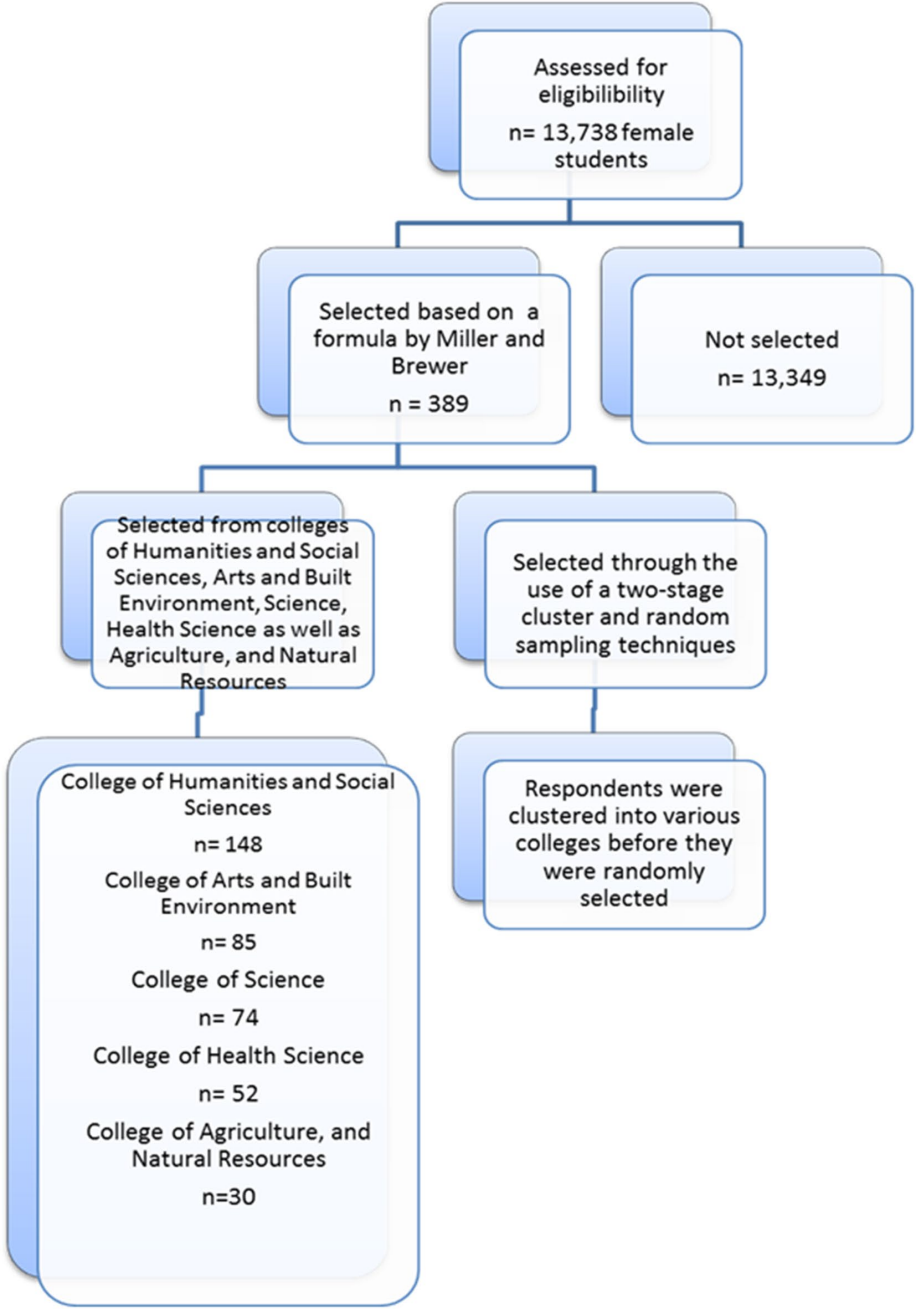
Flow diagram of recruitment of respondents

In each college, the number of respondents was calculated proportionately using the population of the undergraduate females in the various colleges. The respondents were asked to pick pieces of papers that were folded with ‘True’ and ‘False’ options. Those who chose ‘True’ were selected until all the sample size earmarked for each college was obtained. The recruitment of the respondents for the study is shown in Fig. 1.

A closed-ended questionnaire (Additional file 1: Questionnaire) was given to the students during their regular lecture periods. The closed-ended questionnaire was made up of two sections and was written in English. The first section comprised background characteristics of the respondents such as age, religion, ethnicity and income. The second part consisted of information on patterns and prevalence of skin toning practices among the respondents. The Questionnaire included items such as whether or not a respondent has used skin toning products in the last 12 months preceding the survey, the number of times they have used it, the frequency of usage, factors that motivate them to use, the kind of skin toning products they prefer, and the one they mostly used. The questionnaire was explained to the respondents by three trained research assistants recruited from the Department of Geography and Rural Development, KNUST. However, the data collection process was monitored by the fourth author who has a background in Medical Geography as well as Health and Development. To help check call-backs problems, the distribution, and collection of the questionnaires were done by hand and on the same day. This helped to ensure a 100% response rate in the study. The completion of each questionnaire lasted 40 min on the average. Also, written informed consent was obtained from respondents before they were recruited for the study. They were also assured that the information they provided would be treated with absolute confidentiality.

Inferential analytical tools embedded in Statistical Package for the Social Sciences software (version 16) (SPSS) was employed to establish an association between socio-demographic characteristics of the respondents and the use of skin toning products with a significant level of 0.05 or less.

## Results

### Socio-demographic characteristics of respondents

Data gathered on the demographic characteristics of respondents were presented in Table 1. Observed from Table 1, the mean age was 22 ± 1.5 years, and the majority (91%) were single. A little over half (59.1%) were categorized as dark-skinned while the majority (86.6%) grew up in an urban setting. Respondents were selected from level 100 (29.6%), level 200 (34.7%) as well as level 300 (11.6%) and level 400 (24.2%), residing on campus (50.6%) and off-campus (49.4%). Majority of the respondents were Akans (77.9%), and pursuing health-related programmes (76.3%).

**Table 1:** Socio-demographic characteristics of respondents

Variable	Category	N = 389	%	
Age	Less than 20	133	34.2	
	20	63	16.2	
	21	72	18.5	
	22	87	22.4	
	23	13	3.3	Mean ± SD
	24	9	2.3	22 ± 1.5
	25 and above	12	3.1	
Marital status	Single	356	91.5	
	Married	33	8.5	
Skin colour	Fair	159	40.9	
	Dark	230	59.1	
Which area did you grow up	Rural	52	13.4	
	Urban	337	86.6	
Where do you reside during vacation	Rural	64	16.5	
	Urban	325	83.5	
Nature of senior high school attended	Private	39	10.0	
	Public	350	90.0	
Category of senior high school attended.	Mixed school	114	29.3	
	Girls school	275	70.7	
Level of students	Level 100	115	29.6	
	Level 200	135	34.7	
	Level 300	45	11.6	
	Level 400	94	24.2	
Where do you reside in the school	Campus	197	50.6	
	Off-campus	192	49.4	
Classification of accommodation	Hostel	311	79.9	
	Homestel	78	20.1	
Income (Ghs)	Less than 100	73	18.8	
	101–200	119	30.6	
	201–300	67	17.2	
	301–400	36	9.3	
	401–500	24	6.2	Mean ± SD
	More than 500	70	18.0	250.5 ± 1.74
Religion	Christianity	362	93.1	
	Islam	12	3.1	
	Traditional	15	3.9	
Ethnicity	Akan	303	77.9	
	Ewe	41	10.5	
	Ga	3	0.8	
	Mole Dangbani	15	3.9	
	Guan	9	2.3	
	Other specify	18	4.6	
Programme of study	Health-related	297	76.3	
	Non-health related	92	23.7	

### Prevalence and patterns of skin toning practices

On the prevalence and patterns of skin toning practices among female university students (Additional file 2: Table S1), it was revealed that less than half of the study population (40.9%) had practised skin toning within the last 12 months.

The highest proportion of the respondents (51.3%) used skin toning products to treat a skin disorder. Moreover, Creams (38.9%) and Soap or Gel (35.5%) were the skin toning products mostly used by the respondents.

### Socio-demographic factors associated with skin toning practices

In the multivariate analysis, the results show that respondents aged 21 years were 0.4 times more likely to use skin toning products (AOR **=** 0.400, CI 0.121–1.320). Respondents who had dark skin were 3.3 times more likely to use skin toning products (AOR = 3.287, CI 1.503–7.187**)**. Those who attended public school were 1.9 times more likely to use skin toning products (AOR = 1.9, CI 1.1–3.56) and those who attended girls school were 10.7 times more likely to use skin toning products (AOR = 10.764, CI 4.2–27.3). Furthermore, those who were in level 400 were 49 times more likely to use skin toning products (AOR = 49.327, CI 8.48–286.9) and those who received more than 500 cedis were 2 times more likely to use skin toning products (AOR = 2.118, CI 0.419–10.703) as shown in Table 2.

**Table 2:** Socio-demographic factors predicting the practice of skin toning products

Variable	COR (95% CI) Bi-variate analysis	AOR (95% CI) Multi-variate analysis
Age		
Less than 20	1.00	
20	0.680 (0.372–1.242)*	0.491 (0.204–1.184)
21	2.073 (1.116–3.849)*	0.400 (0.121–1.320)*
22	1.305 (0.751–2.266)	0.712 (2.27–2.234)
23	.354 (0.104–1.208)	0.001 (0.000–0.011)
24	0.06 (0.001–0.005)	0.027 (0.002–0.007)
25 and above	1.595 (0.458–5.555)	3.718 (0.467–6.12)
Marital status		
Single	1.00	1.00
Married	0.045 (0.232–0.511)	1.592 (3.98–6.368)
Skin colour		
Fair	1.00	1.00
Dark	2.7 (1.398–3.203)*	3.287 (1.503–7.187)*
Family physician		
Yes	1.00	1.00
No	0.574 (1.393–3.86)	1.415 (0.646–3.097)
Where respondents grew up		
Rural	1.00	1.00
Urban	0.255 (0.934–3.43)*	2.132 (4.46–10.185)
Nature of school attended		
Private	1.00	1.00
Public	2.998 (0.72–15.06)*	1.9 (1.1–3.56)*
Category of school attended		
Mixed school	1.00	1.00
Girls school	6.72 (3.410–15.06)*	10.764 (4.2–27.3)*
Level of student		
Level 100	1.00	1.00
Level 200	0.467 (0.280–0.778)*	10.764 (4.23–27.336)*
Level 300	0.267 (1.129–0.553)*	0.035 (0.006–0.191)*
Level 400	2.416 (1.261–4.630)*	49.327 (8.48–286.9)*
Income (GHS)		
Less than 100	1.00	1.00
101–200	0.619 (0.331–1.157)*	0.144 (0.05–0.412)*
201–300	0.348 (0.173–0.699)*	1.2 (0.8–1.63)*
301–400	0.404 (0.177–0.923)*	0.346 (0.09–1.328)*
401–500	0.166 (0.060–0.459)*	0.001 (0.000–0.006)*
More than 500	1.01 (0.489–2.085)*	2.118 (0.419–10.703)*
Religion		
Christianity	1.00	1.00
Islam	2.005 (0.534–7.530)	0.002 (0.001–0.007)
Traditional	0.243 (0.076–0.778)	0.219 (0.012-4.155)
Ethnicity		
Akan	1.00	1.00
Ewe	2.983 (1.475–6.034)*	21.893 (2.469–194.315)
Ga	1.641 (0.769–3.502)	7.148 (1.211–42.211)
Northerner	0.003 (0.001–0.008)	0.04 (0.002–0.009)
Programme		
Health-related	1.00	1.00
Non-health related	0.891 (0.554–1.434)	1.907 (0.832–4.368)

*CI* confidence interval, *COR* crude odd ratio, *AOR* adjusted odd ratio, *1.00* reference

* *p* < 0.05

Socio-demographic characteristics and the practice of skin toning can be found in Additional file 3: Table S2.

## Discussion

This study examined the prevalence, patterns and factors associated with skin toning practices among female university students in Ghana. To the best of our knowledge, this is one of the first studies in Ghana to provide a detailed understanding of skin toning practices among undergraduate female university students. Fokuo [15] was of the view that the Ghanaian society values good skin colour and it serves as a form of social capital for women especially. In this way one’s self-worth, esteem and standard increases when one has light skin and therefore making light-skinned women the preferred choice in terms of marriage. Because marriage is well thought-out as the ultimate accomplishment within the Ghanaian community, women are, therefore, compelled to improve their skin tone to attract men at all costs. It was expected therefore that, the majority of the respondents of this study would have practiced skin toning within the last 12 months. However, less than half of the respondents (40.9%) had practiced skin toning within the last 12 months, and the higher proportion had done it once (40.9%). Also, about a third of the respondents (34.6%) used skin toning products once in a while, and the highest proportion of the respondents (51.3%) used skin toning products to treat a skin disorder. This suggests that obtaining a smooth and perfect complexion is paramount among women.

Similarly, Ajose [16], as well as Blay [3], reported that people were motivated to tone their skin to improve its appearance. Mpengesi and Nzuza [3] reported that skin toning is seen as a practice to beautify the skin by people determined to improve their appearance and that about 63.3% of people usually tone when they want to eliminate rashes so they will look beautiful. Also, Ajose [16] reported that people tone when they want a smooth complexion or want to clear their skin of any skin disorder. Due to this, de Souza [17] indicated that smooth skin is one of the benefits of toning because everyone admires an even-toned skin without any blemish. Hunter [18], reported that light-skinned African Americans and Mexican Americans as opposed to dark skinned ones had more advantage when it comes to educational opportunities and receiving more income. Hence, being light skinned is the ultimate [19] due to its numerous benefits. The value for lightness is entrenched in the social structures of families and societies at large, thus perpetuating colour hierarchies. This study and the existing literature exhibit the value attached to having an ecstatic, evenly toned and faultless skin complexion which is seen as attractive, and therefore, commendable. This could stimulate others to use all conceivable avenues to attain such a revered attribute.

The study found that respondents who had dark skin, attended public school, went to girls’ school, were in level 400 and received more than GH 500 cedis were significantly more likely to practice skin toning. The findings related to attending public and girls’ schools are relatively new in the existing literature. Our findings contradict with the observation of Hamed et al. [20] that people with coloured skin have an increased prevalence of using skin toning products. The difference in the finding may be attributed to the setting and the methodological differences. Further, we found that the use of skin toning products increases as the level of education of an individual also increases similar to what has been reported previously [20].

## Conclusion

This study examined the prevalence and patterns of skin toning practices among undergraduate female university students at KNUST in Ghana. Less than half of the respondents (40.9%) had practiced skin toning within the last 12 months prior to the survey. Age, skin colour, nature of school attended, type of school attended, level of student and monthly income significantly influence the use of skin toning products among university students in Ghana. We, therefore, argue that policy interventions that seek to reduce skin toning practices among university students should consider micro and broader socio-demographic factors.

## Limitations

The study was limited to the views of female university students; however, the inclusion of male university students could have paved the way for new findings. Further, the use of one institution and the period within which data was collected limits the extent to which the findings could be generalized. It is, therefore, recommended that future research should be extended to students in other universities and also consider the views of male students on the use of skin toning products.

## Additional files

**Additional file 1.** Questionnaire.

**Additional file 2: Table S1.** Prevalence and patterns of skin toning practices among female university students.

**Additional file 3: Table S2.** Socio-demographic characteristics influencing the practice of skin toning.

## Data Availability

All data generated or analysed during this study are included in this published article (and its additional information files). A complete document of this study and its results can also be found at the Library of KNUST, Kumasi.
