# Supplementary material for: Prevalence and patterns of skin toning practices among female students in Ghana: a cross-sectional university-based survey
Source: BMC Res Notes. 2019 May 28;12:299. doi: 10.1186/s13104-019-4327-8 (PMC6537155; doi:10.1186/s13104-019-4327-8)
Supplement: Supplementary file 1 — Additional file 1. Questionnaire. [file 13104_2019_4327_MOESM1_ESM.docx]

Questionnaire number………………………………………………………………………………

Date………………………………………………………………………………………..………..

Programme …………………………………………………………………………………………

**SECTION 1: SOCIO-DEMOGRAPHIC CHARACTERISTICS OF RESPONDENT**

**1.** Age (years)

1. less than 20 [ ] 2. 20 [ ] 3. 21 [ ] 4. 22 [ ] 5. 23 [ ]

6. 24 [ ] 7. 25 and above [ ]

**2**. Marital status

1. Single/Widow/Widower/ Divorced [ ] 2. Married/Cohabitated [ ]

**3.** Skin Complexion/colour of skin

1. Fair skin [ ] 2. Dark skin [ ]

**4.** Do you live with your parents?

1. Yes [ ] 2. No [ ]

**5.** Do you have family physician?

1. Yes [ ] 2. No [ ]

**6.** Which area did you grow up?

1. Rural [ ] 2. Urban [ ]

**7.** Where do you reside during vacation?

1. Rural [ ] 2. Urban [ ]

**8.** Nature of Senior High School attended

1. Private [ ] 2. Public [ ]

**9.** Category of Senior High School attended

1. Mixed school [ ] 2. Girls’ school [ ]

**10.** Level of Student

1. Level 100 [ ] 2. Level 200 [ ] 3. Level 300 [ ] 4. Level 400 [ ]

**11**. Where do you reside in school?

1. Campus [ ] 2. Off-campus [ ]

**12.** Classification of Accommodation

1. Hostel [ ] 2. Homstel [ ]

**13.** Total income per Month

1. Less than GHS 100 [ ] 2. GHS 101-200 [ ] 3. GHS 201-300 [ ]

4. GHS 301-400 [ ] 5. GHS 401-500 [ ] 6. More than GHS500 [ ]

**14.** Religious background

1.Christianity [ ] 2. Islam [ ] 3. Traditional [ ]

4. Other, specify……………………………….

**15**. Tribe/Ethnicity

1. Akan [ ] 2. Ewe [ ] 3. Ga-Dangme [ ]

4. Mole-Dagbani [ ] 5. Guan [ ] 6. Other, specify ………………

**16.** Programme of study

1. Science related [ ] 2. Non-Science related [ ]

**SECTION 2: PATTERN OF UTILISATION OF SKIN TONNING COSMETICS**

**17.** Have you used skin toning products before?

1. Yes [ ] 2. No [ ]

**18**. What motivates you to use skin toning products? [ *Tick all that apply*]

1. Lighter skin is more beautiful and looks healthier [ ]

2. Lighter skin provides higher self-esteem [ ]

3. Lighter skin implies belonging to higher social class [ ]

4. Lighter skin helps in getting better job [ ]

5. Treatment of skin disorders [ ]

**19.** How many times have you used skin toning products in the last 12 months?

1.Once [ ] 2. Twice [ ] 3. Thrice [ ] 4. Four times [ ] 5. Five times or more [ ]

**20.** How often do you use skin toning products?

1. Every week [ ] 2.Every two weeks [ ] 3. Every month [ ]

4. Every three months [ ] 5. More than every three months [ ]

**21.** What types(s) of skin toning products do you use? [ *Tick all that apply*]

1. Creams [ ] 2. Soap or Gel [ ] 3. Facial cleanser [ ]

4. Facial moisturizer [ ] 5. Capsules [ ] 6.Pills [ ] 7. Injection [ ]

8. Other, specify………………………

**22.** Which skin toning product do you prefer?

1. Locally manufactured [ ] 2. International product [ ] 3. Both [ ]

**23.** Why do you prefer such products?

…………………………………………………………………………………………………..

**24.** Do you know any of the active ingredients used in skin toning products?

1. Yes [ ] 2. No [ ]

**25.** Where do you purchase the products from? [ *Tick all that apply*]

1.Cosmetics shop [ ] 2. Drug store/Pharmacy shop [ ] 3. Online [ ]

4. Market [ ] 5. Traditional Medical Practitioners [ ]

6. Health Care Practitioners (Hospitals / Clinics) [ ] 7. Beauty store [ ]

**26.** What factor(s) do you consider during purchasing? [ *Tick all that apply*]

1.Brand influence [ ] 2.Reasonable price [ ] 3.Effectiveness of the product within a short period of application [ ] 4. Ingredients used [ ] 5. Advertisement [ ]

6. Friend Recommendation [ ]

**27.** Do you check the ingredients before purchasing?

1.Yes [ ] 2. No [ ]

**28.** How do you learn about these cosmetics? [*Tick all that apply*]

1. Family member [ ] 2. Friend or acquaintance [ ]

3. Literature/ books [ ] 4. Media/ television/radio [ ]

5. From doctor or other health care provider [ ] 6. Other, specify....................

**29.** How much does it cost you to buy the skin toning products?

1. Less than GHS30 [ ] 2. GHS30 – GHS60 [ ] 3. More than GHS60 [ ]

**30.** In general, what percentage of your income do you spend on Skin toning products?

1. Less than 5% [ ] 2. 6% - 10% [ ] 3. 11% - 15% [ ] 4. More than 15% [ ]

**31.** Are skin toning products affordable?

1. Yes [ ] 2. No [ ]

**32.** How would you rate the affordability of skin toning products?

1. Very Affordable [ ] 2. Slightly affordable [ ] 3. Less affordable [ ]

4.Not affordable [ ]
