# Supplementary material for: Prevalence and patterns of skin toning practices among female students in Ghana: a cross-sectional university-based survey
Source: BMC Res Notes. 2019 May 28;12:299. doi: 10.1186/s13104-019-4327-8 (PMC6537155; doi:10.1186/s13104-019-4327-8)
Supplement: Supplementary file 2 — Additional file 2: Table S1. Prevalence and patterns of skin toning practices among female university students. [file 13104_2019_4327_MOESM2_ESM.docx]

**Table S1: Prevalence and patterns of skin toning practices among female university students**

| **Variable** | **Category** | **N=389** | | **%** |
| --- | --- | --- | --- | --- |
| **Have you practised skin toning in the last 12 months** | Yes | 159^a^ | 40.9 | |
|  | No | 240 | 59.1 | |
| **How many times have you used skin toning products in the last 12 months** | Once | 65 | 40.9 | |
|  | Twice | 26 | 16.4 | |
|  | Thrice | 27 | 17.0 | |
|  | Four times | 5 | 3.1 | |
|  | Five times or more | 36 | 22.6 | |
|  | Every week | 54 | 34.0 | |
|  | Every two weeks | 41 | 25.8 | |
| **How often do you use skin toning products** | Every month | 8 | 5.0 | |
|  | Every three months | 1 | .6 | |
|  | Once in a while | 55 | 34.6 | |
| **Which of the following factors motivate you to use skin toning products** ^b^ | Lighter Skin is more beautiful and healthier | 41 | 21.9 | |
|  | Lighter skin gives self-esteem | 21 | 11.2 | |
|  | Belonging to the higher social class | 5 | 2.7 | |
|  | Lighter skin helps one to secure jobs | 24 | 12.8 | |
|  | Helps to treat a skin disorder | 96 | 51.3 | |
|  | Locally manufactured | 28 | 17.6 | |
| **Which skin toning products do you prefer** | International product | 91 | 57.2 | |
|  | Both | 40 | 25.2 | |
| **Which of the following skin toning products do you use** ^b^ | Creams | 79 | 38.9 | |
|  | Soap or Gel | 72 | 35.5 | |
|  | Facial Cleaner | 21 | 10.3 | |
|  | Capsules | 20 | 9.9 | |
|  | Pills | 8 | 3.9 | |
|  | Injection | 3 | 14.8 | |

^a^ Responses for subsequent questions sum up to 159 with the exception of those indicated as “ ^b”^

^b^ Frequency is more than 159 because multiple responses were applied
