# Supplementary material for: Prevalence and patterns of skin toning practices among female students in Ghana: a cross-sectional university-based survey
Source: BMC Res Notes. 2019 May 28;12:299. doi: 10.1186/s13104-019-4327-8 (PMC6537155; doi:10.1186/s13104-019-4327-8)
Supplement: Supplementary file 3 — Additional file 3: Table S2. Socio-demographic characteristics influencing the practice of skin toning. [file 13104_2019_4327_MOESM3_ESM.docx]

**Table S2: Socio-demographic characteristics influencing the practice of skin toning**

| Variable | Category | |  | |  |  |
| --- | --- | --- | --- | --- | --- | --- |
|  |  |  | N=389 | % | Chi Square | P-value |
| **Age** | | Less than 20 | 133 | 34.2 | 21.390 | 0.002 |
|  |  | 20 | 63 | 16.2 |  |  |
|  |  | 21 | 72 | 18.5 |  |  |
|  |  | 22 | 87 | 22.4 |  |  |
|  |  | 23 | 13 | 3.3 |  |  |
|  |  | 24 | 9 | 2.3 |  |  |
|  |  | 25 and above | 12 | 3.1 |  |  |
| **Marital status** | | Single | 356 | 91.5 | 4.162 | 0.041 |
|  |  | Married | 33 | 8.5 |  |  |
| **Skin color** | | Fair | 159 | 40.9 | 12.736 | 0.001 |
|  |  | Dark | 230 | 59.1 |  |  |
| **Do you have a family physician** | | Yes | 117 | 30.1 | 0.244 | 0.621 |
|  |  | No | 272 | 69.9 |  |  |
| **Which area did you grow up** | | Rural | 52 | 13.4 | 4.834 | 0.028 |
|  |  | Urban | 337 | 86.6 |  |  |
| **Where do you reside during vacation** | | Rural | 64 | 16.5 | 0.104 | 0.747 |
|  |  | Urban | 325 | 83.5 |  |  |
| **Nature of senior high school attended** | | Private | 39 | 10.0 | 26.742 | 0.001 |
|  |  | Public | 350 | 90.0 |  |  |
| **Type of senior high school attended** | | Mixed school | 114 | 29.3 | 12.182 | 0.001 |
|  |  | Girls school | 275 | 70.7 |  |  |
| **Level of students** | | level 100 | 115 | 29.6 | 43.024 | 0.001 |
|  |  | level 200 | 135 | 34.7 |  |  |
|  |  | level 300 | 45 | 11.6 |  |  |
|  |  | level 400 | 94 | 24.2 |  |  |
| **Where do you reside in the school** | | Campus | 197 | 50.6 | 0.012 | 0.914 |
|  |  | off-campus | 192 | 49.4 |  |  |
| **Classification of accommodation** | | Hostel | 311 | 79.9 | 2.761 | 0.251 |
|  |  | Homestel | 78 | 20.1 |  |  |
| **Income (Ghs)** | | Less than 100 | 73 | 18.8 |  |  |
|  |  | 101-200 | 119 | 30.6 |  |  |
|  |  | 201-300 | 67 | 17.2 |  |  |
|  |  | 301-400 | 36 | 9.3 | 23.642 | 0.001 |
|  |  | 401-500 | 24 | 6.2 |  |  |
|  |  | More than 500 | 70 | 18.0 |  |  |
| **Religious background** | | Christianity | 362 | 93.1 | 7.891 | 0.019 |
|  |  | Islam | 12 | 3.1 |  |  |
|  |  | Traditional | 15 | 3.9 |  |  |
| **Ethnicity** | | Akan | 303 | 77.9 |  |  |
|  |  | Ewe | 41 | 10.5 |  |  |
|  |  | Ga | 3 | 0.8 |  |  |
|  |  | Mole Dangbani | 15 | 3.9 | 25.075 | 0.000 |
|  |  | Guan | 9 | 2.3 |  |  |
|  |  | Other | 18 | 4.6 |  |  |
| **Programme of study** | | Health-related | 297 | 76.3 | 0.225 | 0.636 |
|  |  | Non Health-related | 92 | 23.7 |  |  |

* *p ˂ 0.05.*
